# Sustained Muscle Deoxygenation vs. Sustained High VO_2_ During High-Intensity Interval Training in Sprint Canoe-Kayak

**DOI:** 10.3389/fspor.2019.00006

**Published:** 2019-07-31

**Authors:** Myriam Paquette, François Bieuzen, François Billaut

**Affiliations:** ^1^Département de kinésiologie, Université Laval, Quebec, QC, Canada; ^2^Institut National du sport du Québec, Montreal, QC, Canada

**Keywords:** oxygen saturation, peripheral adaptations, aerobic fitness, sprint kayak, sprint canoe, interval training

## Abstract

Recent data suggests that peripheral adaptations, i.e., the muscle ability to extract and use oxygen, may be a stronger predictor of canoe-kayak sprint performance compared to VO_2_max or central adaptations. If maximizing the time near VO_2_max during high-intensity interval training (HIIT) sessions is believed to optimize central adaptations, maximizing the time near maximal levels of muscle desaturation could represent a critical stimulus to optimize peripheral adaptations.

**Purpose:** Therefore, the purpose of this study was to assess the VO_2_, muscle oxygenation and cardiac output responses to various HIIT sessions, and to determine which type of HIIT elicits the lowest muscle oxygenation and the longest cumulated time at low muscle O_2_ saturation.

**Methods:** Thirteen well-trained canoe-kayak athletes performed an incremental test to determine VO_2_max and peak power output (PPO), and 4 HIIT sessions (HIIT-15: 40x[15 s at 115%PPO, 15 s at 30%PPO]; HIIT-30: 20x[30 s at 115%PPO, 30 s at 30%PPO]; HIIT-60: 6x[1 min at 130%PPO, 3 min rest]; sprint interval training (SIT): 6x[30 s all-out, 3 min 30 rest]) on a canoe or kayak ergometer. Portable near-infrared spectroscopy monitors were placed on the *Latissimus dorsi* (LD), *Biceps brachii* (BB), and *Vastus lateralis* (VL) during every session to assess changes in muscle O_2_ saturation (SmO_2_, % of physiological range).

**Results:** HIIT-15 and HIIT-30 elicited a longer time >90%VO_2_max (HIIT-15: 8.1 ± 6.2 min, HIIT-30: 6.8 ± 4.6 min), compared to SIT (1.7 ± 1.3 min, *p* = 0.006 and *p* = 0.035) but not HIIT-60 (4.1 ± 1.7 min). SIT and HIIT-60 elicited the lowest SmO_2_ in the VL (SIT: 0 ± 1%, HIIT-60: 8 ± 9%) compared to HIIT-15 (26 ± 12%, *p* < 0.001 and *p* = 0.007) and HIIT-30 (25 ± 12%, *p* < 0.001 and *p* = 0.030). SIT produced the longest time at >90% of maximal deoxygenation in all 3 muscles, with effect sizes ranging from small to very large.

**Conclusions:** Short HIIT performed on a canoe/kayak ergometer elicits the longest time near VO_2_max, potentially conducive to VO_2_max improvements, but SIT is needed in order to maximize muscle deoxygenation during training, which would potentially conduct to greater peripheral adaptations.

## Introduction

High-intensity interval training (HIIT) is considered one of the most effective training for improving performance in athletes from various sports (Buchheit and Laursen, 2013a). HIIT involves alternating short (<45 s) to long (2–4 min) bouts of high-intensity (usually >85% max HR) exercise with recovery periods (Weston et al., 2014). In the early 2000s, little was known on the optimal type of HIIT program for producing the greatest improvement in endurance performance in trained athletes (Laursen and Jenkins, 2002). Since then, a lot of research has focused on understanding the acute and chronic effects of different forms of HIIT to optimize its prescription in athletes.

In 1986, Wenger and Bell (1986) published a review of the literature, where they concluded that VO_2_max enhancement was positively related to exercise intensity, for intensities from 50 to 100% of VO_2_max, and showed that the greatest improvements occurred when training was performed at an intensity between 90 and 100% of VO_2_max. It led several authors to suggest that training near or at VO_2_max is an optimal stimulus to improve VO_2_max (Billat, 2001). From a physiological perspective, training at or near VO_2_max would impose a maximal stress to the physiological processes and structures limiting VO_2_max and should, therefore, promote adaptations in these structures and processes. For example, it has been shown that mechanical overload, the main stimulus for morphological adaptation of the myocardium and hence enhancement of the cardiac output, is maximal when exercising at the intensity eliciting VO_2_max (Åstrand et al., 1964). Thus, there has been great research interest in characterizing time spent at or near (>90%) VO_2_max in different training protocols, to identify the training sessions that would elicit the longest time near VO_2_max. In a literature review published in 2006, Midgley and McNaughton (2006) detailed the training characteristics that produce the longest time at or near VO_2_max. They concluded that, in order to maximize time spent near VO_2_max, work intervals of 15–30 s should be performed at an intensity of 90–105% of the minimal speed eliciting VO_2_max (vVO_2_max), and recovery intervals of 15–30 s should be performed at an intensity between 50% of VO_2_max and lactate threshold.

Sprint interval training (SIT), consisting of low volume all-out supramaximal (at a greater intensity than 100% VO_2_max) sprints of typically 20–30 s duration, interspersed by long (≥2 min) recovery intervals has been shown to improve endurance performance in trained cyclists (Laursen et al., 2002) and runners (Esfarjani and Laursen, 2007). Although time spent at >90% VO_2_max during SIT sessions is low (typically 0–60 s in trained cyclists for an entire training session), it has been suggested that muscle O_2_ demand is high, especially as the number of sprints increases, as suggested by low muscle oxygenation levels (Buchheit et al., 2012a). Therefore, SIT appears to be an optimal training stimulus to improve endurance performance through peripheral (muscular) adaptations. But all-out SIT is associated with high neuromuscular fatigue (Buchheit and Laursen, 2013b), which could limit the ability of the athletes to perform other training sessions. It is currently unknown if supramaximal efforts of lower intensity (120–130% maximal aerobic power) but longer duration (>45 s) could elicit the same peripheral demand, while inducing less neuromuscular fatigue. If so, it would be of great interest for athletes, decreasing the likeliness of interference between HIIT and other training sessions (moderate-intensity continuous training, strength training, speed training, power training, etc.). To our knowledge, acute response to longer supramaximal interval training has not been assessed so far.

In sprint canoe-kayak, Olympic individual events are 200- and 500-m (~38 to ~120 s) for women and 200-m and 1,000-m (~34 to ~220 s) for men. While VO_2_max is often considered a major performance factor in longer distance sprint canoe-kayak events (Michael et al., 2008), results from a recent study suggest that peripheral adaptations (as assessed via near infrared spectroscopy (NIRS) derived changes in muscle oxygenation) may be stronger predictors of canoe-kayak performance in both short and long events (Paquette et al., 2018). In fact, since 200 and 500-m events are performed at an intensity greater than VO_2_max, and since canoe-kayak is an upper body dominant sport, and skeletal muscles in the arms typically display larger cross sectional area of type II muscle fibers, it makes this discipline more reliant on muscle power than cardiorespiratory fitness. Therefore, there is a need to identify training sessions that elicit a high peripheral demand in this sport.

The oncoming of affordable and portable NIRS monitors has increased accessibility to muscle oxygenation measures during exercise. Researchers have used peak VO_2_ and time spent >90% VO_2_max during HIIT sessions to assess the central demand of HIIT, and we suggest that maximal muscle deoxygenation and cumulated time at >90% maximal muscle deoxygenation are good indicators of acute peripheral demand during HIIT sessions.

The purpose of this study was to assess the VO_2_, muscle oxygenation, and cardiac output responses to 4 types of interval training commonly used by canoe-kayak athletes, and to determine which type of interval training elicits the lowest muscle O_2_ levels and the longest cumulated time at low muscle O_2_ saturation.

## Methods

### Subjects

Thirteen canoe-kayak athletes participated in this study, of which 9 were kayakers (3 women, 6 men) and 4 were canoeists (2 women, 2 men). Participants were 22 ± 3 years of age (range 19–27 years old) and weighted 71.5 ± 8.3 kg. Four of them were members of the Canadian National Team in sprint or slalom, and nine were provincial-level athletes in sprint canoe or kayak. This study was approved by the local ethics committee and was conducted in accordance to the principles established in the Declaration of Helsinki, with verbal and written informed consent provided by all participants.

### Experimental Design

Athletes performed a maximal incremental test on a canoe or kayak, for the determination of maximal oxygen consumption (VO_2_max), maximal cardiac output (Qmax), and peak power output (PPO). Next, they completed four interval training sessions on a canoe or kayak ergometer in a randomized order to determine the acute physiological response associated with each interval training type.

### Methodology

#### Maximal Incremental Test

All participants performed a continuous incremental VO_2_max testing consisting of six 2-min stages of increasing intensity on a kayak or canoe ergometer (SpeedStroke Gym, KayakPro, Florida, USA). The ergometer was calibrated before each test, according to the manufacturer recommendations and tension in the ergometer's ropes was verified regularly (Tanner and Gore, 2013). Participants received stroke-by-stroke feedback during the test and were asked to maintain a constant intensity that would elicit effort perception of 2, 4, 5, 6, 8, and 10/10 during stages 1–6, respectively. Stroke rate ranges were given for the first 5 stages for canoe (30–35; 35–40; 40–45; 45–50; 50–55 strokes per min) and kayak (60–65; 65–75; 75–85; 85–95; 95–105 strokes per min) to help athletes select the right intensity in each stage. Power output (PO) was recorded on a computer, using the eMonitorPro2 software (KayakPro, Florida, USA).

#### Interval Training Sessions

The athletes completed four different HIIT sessions on the canoe or kayak ergometer in a randomized order. There was always more than 24 h and less than a week between HIIT sessions and athletes must refrain from doing any intense physical activity for 24 h before the training session. Training sessions are described in Table 1. Athletes were asked to rate their level of exertion and their level of enjoyment on a scale of 1 to 10 at the end of every HIIT session.

**Table 1:** Description of the interval training sessions.

**Session**	**Work duration**	**Work target intensity**	**Recovery duration**	**Recovery target intensity**	**Number of repetitions/set**	**Number of sets**	**Rest between sets**
HIIT-15	15 s	110% PPO	15 s	30% PPO	20	2	5 min
HIIT-30	30 s	110% PPO	30 s	30% PPO	10	2	5 min
HIIT-60	1 min	130% PPO	3 min	Choice	6	1	–
SIT	30 s	All-out	3 min 30	Choice	6	1	–

*PPO, peak power output; Choice: the athletes could rest passively or paddle slowly*.

#### VO_2_ and Cardiac Output

During all sessions, expired air was continuously recorded using a breath-by-breath gas analyzer (Vmax Encore metabolic cart, CareFusion Corp, California, USA). PPO was the average power output on the last 2-min stage of the maximal incremental test. Heart rate (HR) and cardiac output (Q) were evaluated during all tests using thoracic electrical bioimpedance (Physioflow, Manatec Biomedical, France). All devices were calibrated according to manufacturer guidelines before every test. Arteriovenous O_2_ difference [(a-v)O_2_-diff (ml/dl)] was calculated using the following equation: (a-v)O_2_-diff = VO_2_ (L/min)/Q (L/min)^*^100 and stroke volume (SV) was calculated from Q and HR. VO_2_max, maximal Q (Qmax), maximal SV (SVmax), and maximal (a-v)O_2_-diff were defined as the highest values achieved over a 30-s period during the incremental test. Time over 90 and 95% of VO_2_max, Qmax, and HRmax were calculated for all sessions. Peak VO_2_, peak Q and peak HR were also calculated for each session, and defined as the highest 30-s average for VO_2_ and Q and highest 5-s average for HR value reached during the session.

#### Muscle Oxygenation

During all tests, Moxy NIRS monitors (Fortiori Design, Minnesota, USA) were placed on three active muscles: *latissimus dorsi* (LD)—midpoint between the inferior border of the scapula and posterior axillar fold (Borges et al., 2015) –, *biceps brachii* (BB)—middle of the BB muscle belly (8–12 cm above the elbow fold)—and *vastus lateralis* (VL)—distal part of the VL muscle belly (10–15 cm above the proximal border of the patella) (Billaut and Buchheit, 2013). NIRS monitors were placed on the athlete's dominant side for kayakers, and on the front leg and opposite BB and LD for canoeists, parallel to the muscle fiber orientation. They were attached and secured with a double-sided adhesive disk and an adhesive patch, and covered by a dark bandage to reduce the intrusion of extraneous light. The Moxy monitors position was marked on the athlete's skin to ensure the monitors were placed on the same site in every testing session. Skinfold thickness at each site was measured using a skinfold caliper (Harpenden Ltd) to ensure that the skinfold thickness was less than half the distance between the emitter and the detector (25 mm). The raw muscle O_2_ saturation (SmO_2_) signal was treated using a smooth spline filter to reduce the noise created by movement (Rodriguez et al., 2018). During exercise, SmO_2_ represents the balance between O_2_ delivery and extraction by the muscle (Ferrari et al., 2011). Minimum and maximum SmO_2_ were the absolute lowest and highest 5-s average SmO_2_ reached during any of the HIIT session. Minimum and maximum SmO_2_ values were determined from the lowest and highest observed values throughout the entire experiment for each subject. All SmO_2_ values were then normalized, so that 0 and 100% represent these minimum and maximum SmO_2_ of the participant, respectively. SmO_2_ values are presented in these normalized values in the results section and minimum SmO_2_ will be referred as deoxy max thereafter. As for VO_2_max and cardiac output values, time over 90 and 95% of deoxy max were calculated for all HIIT sessions and defined as the time spent at a SmO_2_ value of <10% and <5% in this normalized scale. Time spent above 90 and 95% deoxy max were determined during both work and rest intervals to obtain a complete picture of peripheral metabolic disturbances during the given sessions. Peak muscle deoxygenation (peak deoxy) was calculated for each muscle during each session, and was defined as the lowest 5-s average SmO_2_ reached during the session.

### Statistical Analysis

Means and standard variations were calculated for physiological parameters, and ANOVAs with Tukey *post-hoc* tests were used to assess differences between groups. Cohen's d effect sizes (ES) and 90% confidence intervals were also computed for differences in means between sessions and ES of 0.2, 0.6, 1.2, and 2.0 were considered small, moderate, large, and very large differences, respectively. For the SmO_2_ data, a robust smallest worthwhile change (SWC) anchor is not evident, therefore the SWC was calculated using the standardized mean difference of 0.2 between subject standard deviations (SD).

## Results

### Athlete's Characteristics

Participants had a VO_2_max of 51.8 ± 7.7 ml/kg/min, or 3.7 ± 0.8 L/min, a maximal cardiac output of 25.4 ± 3.5 L/min, a maximal SV of 147 ± 24 ml/beat, a maximal (a-v)O_2_-diff of 14.5 ± 2.0 ml/dl and a maximal heart rate of 194 ± 5 bpm. Their PPO during the incremental test was 122 ± 36 W, or 1.69 ± 0.45 W/kg.

### Cardiorespiratory and Subjective Responses

**Figure 1:**
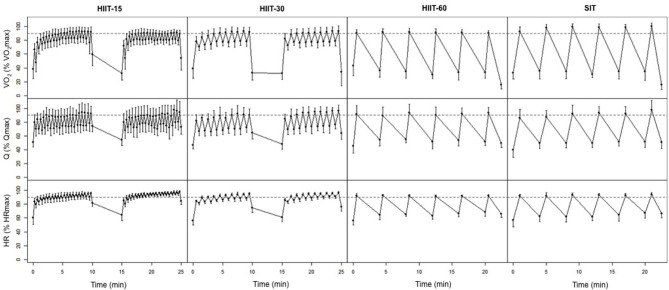
Average VO_2_, Q, and HR at the end of each work and rest interval during the four interval sessions. Horizontal dashed lines represent 90% of VO_2_max, Qmax and HRmax, respectively.

Figure 1 displays the average cardiorespiratory parameters at the end of each work and rest interval for the four training sessions. Table 2 shows the power output maintained, and the VO_2_ and cardiac output responses during each HIIT session. The highest RPE was reached in the SIT session, but it did not differ statistically from the other sessions RPE ratings. VO_2_peak was greater in HIIT-60 compared to HIIT-15 (ES 1.34 [0.62, 2.08], *p* = 0.02), HIIT-30 (ES 1.41 [0.70, 2.14], *p* = 0.01), and SIT (ES 2.32 [1.49, 3.16], *p* < 0.01). Peak Q was not different between sessions. Time spent >90% VO_2_max was higher in HIIT-15 (ES 1.71 [0.94,2.48], *p* < 0.01) and HIIT-30 (ES 1.74 [0.98,2.50], *p* < 0.01) compared to SIT. HIIT-15 and HIIT-30 also elicited a greater cumulated time at >90% Qmax compared to SIT (HIIT-15: ES 1.35 [0.46, 2.25], *p* = 0.04, HIIT-30: ES 1.09 [0.30, 1.88], *p* = 0.02) and greater cumulated time >90% HRmax (HIIT-15: ES 6.39 [4.46, 8.31], *p* < 0.01, HIIT-30: ES 4.19 [2.91, 5.47], *p* < 0.01).

**Table 2:** Power output, VO_2_max and cardiac output values during each interval session.

**Session**	**HIIT-15**	**HIIT-30**	**HIIT-60**	**SIT**
Work intensity (%PPO)	116 ± 16	117 ± 19	133 ± 21	192 ± 36
Recovery intensity (%PPO)	32 ± 12	29 ± 5	18 ± 18	23 ± 23
Session enjoyment (/10)	6.9 ± 2.2	7.0 ± 2.2	6.6 ± 2.4	7.2 ± 2.2
Session RPE (/10)	7.9 ± 1.0	8.1 ± 1.7	7.5 ± 1.8	8.8 ± 0.9
Peak VO_2_ (%VO_2_max)	92 ± 5	91 ± 5^$^	98 ± 4	87 ± 5^$^
Peak HR (%HRmax)	97 ± 3	97 ± 2	96 ± 2	95 ± 2
Peak Q (%Qmax)	95 ± 8	94 ± 6	93 ± 9	90 ± 7
Cumulated time > 90% VO_2_max (min)	8.1 ± 6.2^*^	6.8 ± 4.6^*^	4.1 ± 1.7	1.7 ± 1.3
Cumulated time > 95% VO_2_max (min)	4.9 ± 5.3^*^	3.5 ± 4.3	2.9 ± 1.8	1.0 ± 1.0
Cumulated time > 90% Qmax (min)	5.6 ± 4.4^*^	4.0 ± 2.9^*^	2.0 ± 1.9	1.5 ± 1.6
Cumulated time > 95% Qmax (min)	2.2 ± 2.3	1.8 ± 1.6	1.1 ± 1.3	0.9 ± 1.1
Cumulated time > 90% HRmax (min)	16.6 ± 2.6^*^^$^	13.7 ± 3.3^*^^$^	5.3 ± 2.3	3.6 ± 1.5
Cumulated time > 95% HRmax (min)	7.8 ± 5.5^*^^$^	4.8 ± 3.3^*^	1.6 ± 1.8	0.4 ± 0.8

*PPO, peak power output*.

$ *Different from HIIT-60 (p < 0.05)*.

* *Different from SIT (p < 0.05)*.

### Muscle Oxygenation Response

**Figure 2:**
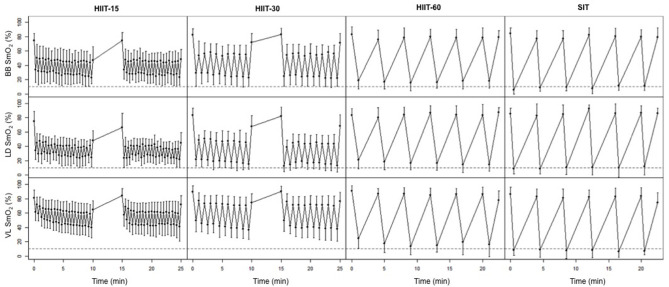
Average SmO_2_ in the BB, LD, and VL at the end of each work and rest interval during the four interval sessions. Horizontal dashed lines represent 10% SmO_2_ (90% of maximal deoxygenation).

Figure 2 displays the average SmO_2_ at the end of each work and rest interval for the four training sessions. Table 3 details the muscle oxygenation response to the 4 HIIT protocols. BB deoxy max was greater in SIT compared to HIIT-15 (ES −1.83 [−1.06, −2.59], *p* = 0.02) and HIIT-30 (ES −1.63 [−0.89, −2.38], *p* = 0.01) and LD deoxy max was greater in SIT compared to HIIT-15 (ES −1.24 [−0.54, −1.95], *p* = 0.04). VL deoxy max was greater in both SIT and HIIT-60 compared to HIIT-15 (SIT: ES 3.86 [−2.77, −4.95], *p* < 0.01, HIIT-60: ES 1.79 [−1.03, −2.55], *p* < 0.01) and HIIT-30 (SIT: ES −3.76 [−2.87, −4.83], *p* < 0.01, HIIT-60: ES −1.70 [−0.95, −2.45], *p* < 0.01). For all 3 investigated muscles, SIT resulted in the longest time spent near maximal muscle deoxygenation compared to session HIIT-15. Specifically in the VL, SIT resulted in the longest time spent at >90 and 95% maximal VL deoxygenation compared to HIIT-15 and HIIT-30, and to a smaller extent to HIIT-60.

**Table 3:** Muscle oxygenation response to the four interval sessions.

**Session**	**HIIT-15**	**HIIT-30**	**HIIT-60**	**SIT**
Peak deoxy BB (%)*^a^*	9 ± 8^*^	10 ± 9^*^	6 ± 7	1 ± 2
Peak deoxy LD (%)*^a^*	9 ± 7^*^	6 ± 7	5 ± 7	2 ± 4
Peak deoxy VL (%)*^a^*	26 ± 12^*^^$^	25 ± 12^*^^$^	8 ± 9	0 ± 1
Cumulated time > 90% deoxy max BB (s)	7.5 ± 8.6^*^	24.7 ± 40.5	24.5 ± 26.8	44.8 ± 40.0
Cumulated time > 90% deoxy max LD (s)	8.7 ± 15.3^*^	29.9 ± 24.9	33.3 ± 37.3	61.2 ± 44.8
Cumulated time > 90% deoxy max VL (s)	0.0 ± 0.0^*^	0.2 ± 0.6^*^	26.5 ± 39.0^*^	83.2 ± 63.1
Cumulated time > 95% deoxy max BB (s)	0.6 ± 1.1^*^	6.8 ± 0.2	5.1 ± 6.0^*^	17.2 ± 13.7
Cumulated time > 95% deoxy max LD (s)	0.7 ± 1.2^*^	6.4 ± 5.7^*^	10.1 ± 12.0	21.3 ± 17.7
Cumulated time > 95% deoxy max VL (s)	0.0 ± 0.0^*^	0.0 ± 0.0^*^	9.1 ± 24.3^*^	46.6 ± 53.7

a *Minimum SmO_2_ during session, presented in normalized units*.

$ *Different from HIIT-60 (p < 0.05)*.

* *Different from SIT (p < 0.05)*.

**Figure 3:**
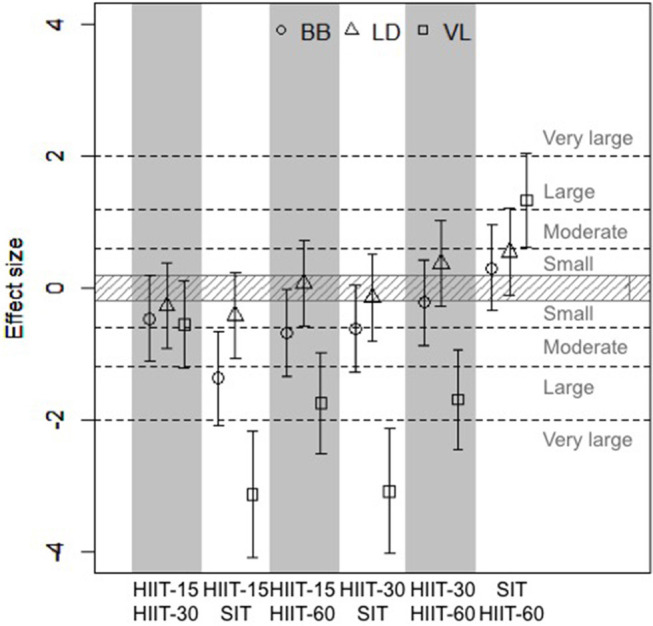
Effect sizes and 90% confidence interval of differences in means between cumulated time spent >90% maximal deoxygenation in the three studied muscles. Positive effect size reflects a greater time spent >90% deoxy max in the first session compared to the second, while negative effect size reflects a smaller time spent >90% deoxy max in the first session compared to the second.

**Figure 4:**
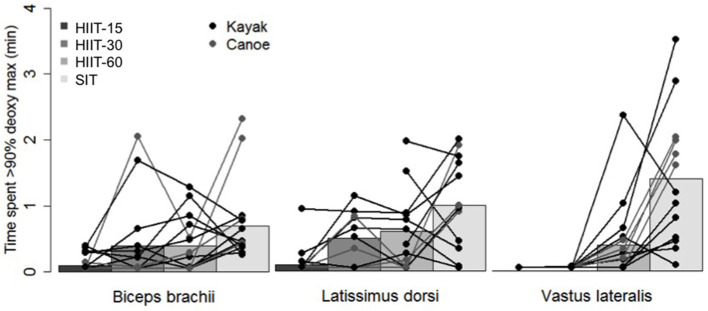
Individual muscle oxygenation response to the four interval sessions.

Figure 3 shows the effect sizes and 90% confidence interval of differences in means between the different sessions for the time spent near maximal muscle deoxygenation. Clear very large differences were found for VL between sessions HIIT-15 and SIT and between sessions HIIT-30 and SIT, and clear large differences were found for VL between sessions HIIT-15 and HIIT-60, HIIT-30 and HIIT-60 and between SIT and HIIT-60 and for BB between sessions HIIT-15 and SIT. Moderate differences were found for BB between HIIT-15 and HIIT-60 and HIIT-30 and HIIT-60. Figure 4 shows the individual response for the time spent near maximal deoxygenation in each HIIT session, for the canoers and the kayakers.

### Difference Between Muscles

Time spent >90% deoxy max was lower in the VL compared to the LD (ES −2.38 [−3.28, −1.48], *p* = 0.04) and tended to be lower compared to BB (ES −1.20 [−1.90, −0.50], *p* = 0.07) in HIIT-30 session. Peak deoxy values were higher in VL compared to BB and LD for both sessions HIIT-15 and HIIT-30 (all *p* < 0.01). There was no difference between investigated muscles for time spent >90% deoxy max or peak deoxy in HIIT-60 and SIT sessions.

## Discussion

Physiological responses to 4 different HIIT sessions were assessed in well-trained canoe-kayak athletes and the most important findings were: (1) short interval training sessions (HIIT-15 and HIIT-30) elicit the most time at high levels of VO_2_, Q and HR, but did not elicit high or sustained levels of muscle deoxygenation; (2) SIT does not elicit much time at high levels of VO_2_, Q or HR, but elicit the highest levels of muscle deoxygenation and the most time spent at high levels of muscle deoxygenation; (3) long supramaximal interval training (HIIT-60) is an hybrid between short intervals and SIT, eliciting higher muscle deoxygenation than short intervals in the VL, and higher VO_2_peak than SIT.

### HIIT and Central Demand

With no surprise, the short interval sessions elicited the highest time >90% VO_2_max compared to the other interval sessions. For short intervals, the ratio of time >90% VO_2_max on total exercise time has been shown to be ~30% (Buchheit and Laursen, 2013a), which is in line with our results (41% for HIIT-15 and 34% for HIIT-30). The lower ratio for HIIT-30 compared to HIIT-15 in our study might be explained by the relatively low intensity of recovery intervals (~30% PPO), which has been associated with reduced time >90% VO_2_max when recovery intervals are >20 s (Buchheit and Laursen, 2013a). The intensity of the recovery intervals was lower than what is usually recommended in short interval sessions, but was the highest intensity the athletes could practically maintain between work intervals, since the resistance from the ergometer elastics creates a high effort perception, even at low workloads. However, the lower intensity in the recovery intervals was compensated by a slightly higher intensity in the work intervals (~115% PPO).

It has been suggested that about 10 min must be spent around VO_2_max in order to elicit important cardiovascular adaptations in endurance athletes (Buchheit and Laursen, 2013a). With 8.1 min and 6.8 min, HIIT-15 and HIIT-30 sessions both come near this recommendation. Since RPE was not maximal at the end of the sessions (~8/10), we can speculate that a third set could have been performed, which would have brought the time >90% VO_2_max around 10–12 min.

In the SIT session, athletes reached 87 ± 5% of their VO_2_max and maintained 1.7 ± 1.3 min >90% VO_2_max. These results are in line with a study where trained cyclists performing 6 all-out 30-s sprints, interspersed by 2 min of passive recovery, reached 90 ± 3% VO_2_max and spent 22 ± 21 s (range 0–60 s) >90% VO_2_max (Buchheit et al., 2012a). Since time spent >90% VO_2_max is inversely related to subjects' VO_2_max (Buchheit et al., 2012a), the slightly higher time spent >90% VO_2_max in our study could be due to the generally lower VO_2_max achieved on kayaking compared to cycling exercise (Michael et al., 2008).

The highest VO_2_ values were reached during the long supramaximal interval session (HIIT-60), but athletes only cumulated 4.1 ± 1.7 min >90% VO_2_max during that session, which still represents a high percentage of the 6 × 1-min work intervals. The higher intensity of the session, accelerating VO_2_ kinetics (Hughson et al., 2000), coupled with the longer effort duration allowed for a high VO_2_ to be reached from the first effort bout. More repetitions would have been needed to accumulate more time >90% VO_2_max, but it would likely not be possible to reach 10 min with this session due to its difficulty. It was suggested that spending 5–7 min at >90% VO_2_max would likely be sufficient for team-sports athletes or for maintenance in endurance athletes. Therefore, such a session could be planned for sprint kayakers wishing to develop 500-m specific speed while maintaining VO_2_max.

Hence, as pointed out by other authors (Midgley and McNaughton, 2006; Buchheit and Laursen, 2013a), short intervals are effective for accumulating training time near VO_2_max and would therefore be a good choice when the goal is to increase VO_2_max.

### HIIT and Peripheral Demand

It has been suggested that repeated fluctuations of muscular O_2_ consumption during training sessions is necessary for muscular oxidative capacity adaptations (Daussin et al., 2008a,b), and that low O_2_ partial pressure at the muscle level is needed to induce mitochondrial biogenesis (Hoppeler et al., 2003). Hence, training sessions that elicit repeated high levels of muscle deoxygenation might present an adequate stimulus to foster peripheral adaptations. Understanding the degree of muscle deoxygenation that occurs during training is therefore important, as it is likely that localized O_2_ availability and extraction triggers the cascade of signals responsible for the marked metabolic adaptations witnessed after high-intensity exercise (Coffey and Hawley, 2007). Muscle deoxygenation during both effort and rest periods were considered in this study, to obtain a complete picture of peripheral metabolic disturbances during the given sessions.

The short interval sessions were the ones eliciting the least deoxygenation and the lowest time spent at high levels of muscle deoxygenation. HIIT-60 elicited a higher muscle deoxygenation than short interval sessions in the VL, but did not elicit a greater time at high deoxygenation levels. SIT was the most effective session to elicit both high and sustained levels of muscle deoxygenation. This is in line with Buchheit et al. (2012a) study, showing muscle deoxygenation during six 30-s all-out efforts to be similar to values observed during maximal voluntary leg contractions or occlusion, known to produce near-to-maximal muscle desaturation. Our results also suggest that effort bout intensity is the most important factor determining the time spent at high levels of muscle deoxygenation. This is in line with results from Stöcker et al. (2015), where a higher cycling intensity was associated with greater muscle deoxygenation. The duration of the work intervals would also have an impact on the degree of muscle deoxygenation. For example, repeated 4-s sprints would reduce SmO_2_ by about 12% (Buchheit et al., 2012b), compared to a reported reduction of 27% for longer sprints (30 s) (Buchheit et al., 2012a).

NIRS SmO_2_ value represents the balance between O_2_ delivery and extraction within the muscle (Ferrari et al., 2011). Thus, a decreased SmO_2_ value could be due to both decreased O_2_ delivery and/or increased O_2_ extraction by the muscle. In supramaximal efforts, it is likely that strong muscle contraction limit O_2_ delivery (Bhambhani, 2004), as suggested by decreased total hemoglobin concentration during SIT (Jones et al., 2015). But deoxyhemoglobin concentration also increased during SIT, suggesting that an increase in muscle O_2_ extraction also occurs (Jones et al., 2015). We chose to only present SmO_2_ here, since recent studies have found that the Moxy monitor provides credible and reliable SmO_2_ values (McManus et al., 2018), but that total hemoglobin values had low variation during exercise and were probably not a valid indicator of blood volume (Crum et al., 2017).

Taken together, these results suggest that SIT elicits a high muscle O_2_ demand, and while the optimal amount of time spent at high muscle deoxygenation in training is not known, it appears that SIT would be an ideal training session to elicit peripheral adaptations. These findings are in line with previous studies showing various peripheral adaptations following SIT (Gibala and McGee, 2008) and a study where muscle deoxygenation was increased in hockey players during SIT following 6 sessions of cycling SIT, potentially indicative of an increase muscle O_2_ extraction capacity with SIT (Jones et al., 2015).

We suggested that HIIT-60 session would represent a good stimulus to improve peripheral adaptations without requiring a maximal effort from the athletes. However, we found that even though maximal deoxygenation was not different from SIT session, time spent at high deoxygenation levels tended to be lower compared to SIT. This is explained by the fact that maximal deoxygenation was only reached near the end of the 1-min work intervals at 130% PPO. We could hypothesize that increasing the intensity during the first 10–15 s of the HIIT-60 work intervals could induce a faster deoxygenation and potentially higher time spent near maximal deoxygenation. Also, HIIT-60 allowed for greater time spent at high deoxygenation level compared to sessions HIIT-15 and HIIT-30 only for the VL, and therefore would be a good hybrid between high central demand and high peripheral demand sessions only for this muscle.

Upper-body muscles deoxygenated to a greater extent in short interval sessions compared to the lower-body muscle investigated. These differences between muscle groups may be explained by a difference in contribution of these muscles. It is possible that upper-body muscles contribute more compared to lower-body muscles to the effort; however, this difference seems to dissipate at higher intensities (>130% PPO). This difference may also come from the lower oxidative capacity of upper body motor units compared to leg muscles (Bhambhani et al., 1998).

We hypothesized that SIT would be an optimal stimulus for peripheral adaptations, but that it would be associated with a higher effort perception. However, while RPE rating was higher following the SIT session compared to the other HIIT sessions, the difference did not reach statistical significance (*p* = 0.08). Session enjoyment was thus similar in all 4 HIIT sessions, suggesting athletes' compliance would not be an issue for any of these sessions (Scanlan et al., 1993). More research is needed to understand the effect of HIIT type on fatigue and recovery.

Even though interval sessions were not matched for interval duration (effort duration varied from 180 to 600 s between sessions), central and peripheral responses were compared between sessions using absolute time-derived variables. Indeed, training sessions were not matched by duration or energy expenditure, but by effort, which is how coaches prescribe training sessions in the field. So if physiological adaptations are driven by the time spent in a given acute physiological state, we believe using typical training sessions and looking at absolute time-derived variables is the best approach in an applied sport research context. Mixing canoeing and kayaking in this study is a potential limitation, given that muscle activity differs between disciplines.

#### Practical Applications

The present study highlights the different acute physiological responses to various types of HIIT. Coaches and athletes who wish to improve VO_2_max through central adaptations should include short intervals to their program. In canoe-kayak, and likely in other upper-body dominant sports, peripheral adaptations are associated with performance and, therefore, SIT sessions targeting the muscle oxidative adaptations should also be included in the training program. Longer supramaximal intervals could be used to train race specific speed while still stimulating VO_2_max, but should probably be modified in order to really target peripheral adaptations.

## Conclusions

Time spent near VO_2_max is high during short-interval HIIT sessions performed on a canoe or kayak ergometer. However, all-out 30-s sprints are required to elicit high and sustained levels of muscle deoxygenation. These findings regarding the acute physiological changes associated with different types of HIIT give insights on potential physiological adaptations following different HIIT protocols. Future studies should assess the chronic effect of HIIT and SIT sessions to ascertain whether time spent near VO_2_max and time spent near maximal muscle deoxygenation are important determinants of VO_2_max and maximal deoxygenation adaptations, respectively, and performance in canoe-kayak athletes.

## Data Availability

The datasets generated for this study are available on request to the corresponding author.

## Ethics Statement

This study was carried out in accordance with the recommendations of Ethics Committee for Health Sciences research of Laval University with written informed consent from all subjects. All subjects gave written informed consent in accordance with the Declaration of Helsinki. The protocol was approved by the Ethics Committee for Health Sciences research of Laval University.

## Author Contributions

All authors listed have made a substantial, direct and intellectual contribution to the work, and approved it for publication.

### Conflict of Interest Statement

The authors declare that the research was conducted in the absence of any commercial or financial relationships that could be construed as a potential conflict of interest.
